# Probing extracellular Sonic hedgehog in neurons

**DOI:** 10.1242/bio.019422

**Published:** 2016-07-07

**Authors:** Erez Eitan, Ronald S. Petralia, Ya-Xian Wang, Fred E. Indig, Mark P. Mattson, Pamela J. Yao

**Affiliations:** 1 Laboratory of Neurosciences, National Institute on Aging Intramural Research Program, Baltimore, MD 21224, USA; 2 Advanced Imaging Core, NIDCD/NIH, Bethesda, MD 20892, USA; 3 Confocal Imaging Facility, Laboratory of Clinical Investigation, National Institute on Aging Intramural Research Program, Baltimore, MD 21224, USA

**Keywords:** Sonic hedgehog, Hippocampal neurons, Extracellular vesicle, Filopodia

## Abstract

The bioactivity of Sonic hedgehog (Shh) depends on specific lipid modifications; a palmitate at its N-terminus and a cholesterol at its C-terminus. This dual-lipid modification makes Shh molecules lipophilic, which prevents them from diffusing freely in extracellular space. Multiple lines of evidence indicate that Shh proteins are carried by various forms of extracellular vesicles (EVs). It also has been shown, for instance, that in some tissues Shh proteins are transported to neighboring cells directly via filopodia. We have previously reported that Shh proteins are expressed in hippocampal neurons. In this study we show that, in the hippocampus and cerebellum of postnatal day (P)2 rats, Shh is mostly found near or on the membrane surface of small neurites or filopodia. We also examined cultured hippocampal neurons where we observed noticeable and widespread Shh-immunolabeled vesicles located outside neurons. Through immunoelectron microscopy and biochemical analysis, we find Shh-containing EVs with a wide range of sizes. Unlike robust Shh activity in EVs isolated from cells overexpressing an N-terminal Shh fragment construct, we did not detect measurable Shh activity in EVs purified from the medium of cultured hippocampal neurons. These results suggest the complexity of the transcellular Shh signaling mechanisms in neurons.

## Abstract (precis)

**Summary:** In the developing brain, Sonic hedgehog molecules localize in neuronal filopodia and preferentially position at cell-to-cell contacts. In cultured neurons, Sonic hedgehog is also found in extracellular vesicles.

## INTRODUCTION

Sonic hedgehog (Shh) signaling pathway plays critical roles in embryonic development as well as in adult tissue homeostasis (Briscoe and Thérond, 2013; Ferent and Traiffort, 2015). In Shh-producing cells, Shh is first synthesized as a 45-kDa precursor protein (Chang et al., 1994; Lee et al., 1994). Following proteolytic cleavage, the 19-kDa N-terminal fragment of Shh undergoes a unique type of modification – with a palmitate added to its N-terminus (Pepinsky et al., 1998) and a cholesterol to its C-terminus (Porter et al., 1996). This dual lipid-modified N-terminal fragment is the signaling molecule that activates the Shh signaling pathway in Shh-responding cells (Chamoun et al., 2001; Lee and Treisman, 2001).

Several mechanisms have been proposed for the movement of the lipid-modified hydrophobic Shh molecules from their producing cells to their responding cells (Thérond, 2012). One of these mechanisms is the use of extracellular vesicles (EVs) as carriers. In *Caenorhabditis* *elegans*, although lacking a Hedgehog (Hh) homolog (Aspöck et al., 1999; Kuwabara et al., 2000), Hh-related proteins are found in small EVs (exosomes) derived from the apical plasma membrane (Liégeois et al., 2006). In *Drosophila* wing imaginal discs, Hh is released in exosomes using an ESCRT (the endosomal sorting complex required for transport)-dependent mechanism (Matusek et al., 2014). In embryonic mouse ventral node, Shh is found to be carried by and moved with vesicular structures that are in a size range of 0.3-5 µm (Tanaka et al., 2005). Recently, it has been reported that EVs with different compositions harbor Shh (Vyas et al., 2014).

In addition to the extracellular vesicular carriers, Shh has been shown to reach their target cells through actin-based filopodia structures, cytonemes (Kornberg and Roy, 2014). In *Drosophila* germline stem cells (Rojas-Ríos et al., 2012), the wing disc and the abdominal epidermis (Bischoff et al., 2013), Hh protein is seen dotted along thin cytonemes extending from Hh-producing cells. Cytoneme formation from these cells directly correlates with Hh concentration in the extracellular space of target areas, implying that cytoneme-mediated Hh transport plays a role in Hh spreading (Rojas-Ríos et al., 2012; Bischoff et al., 2013). A Shh signal also can be transmitted through cytoneme-to-cytoneme contacts. For example, in chick limb bud, Shh particles are found traveling along thin long cytonemes from their producing cells in the direction towards Shh responding cells (Sanders et al., 2013). The Shh-containing cytonemes make stable contacts with cytonemes extended from Shh responding cells, which house co-receptors for Shh (Sanders et al., 2013). Thus, these findings suggest that transmitting Shh signals from one cell to another can occur through direct cell-to-cell contacts.

In the brain, Shh is found in multiple types of neurons (Traiffort et al., 1998; Wallace, 1999; Garcia et al., 2010; Petralia et al., 2011a; Gonzalez-Reyes et al., 2012; Harwell et al., 2012). In the cerebral cortex, Shh produced by layer V corticofugal neurons signals its presynaptic partners – the incoming projection neurons of layer II/III – to form synaptic contacts (Harwell et al., 2012). In the developing cerebellum, Shh produced by Purkinje cells stimulates the growth of granule neuron precursor cells (Wechsler-Reya and Scott, 1999; Wallace, 1999). In the mature cerebellum, neuron-derived Shh continues to function by determining the molecular features of neighboring glial cells (Farmer et al., 2016). While these findings collectively indicate paracrine and juxtacrine Shh signaling in the brain, how neuron-derived Shh is conveyed to its target cells remains largely unknown. In this study, we investigated extracellular Shh *in vivo* in the developing hippocampus and cerebellum and *in vitro* in primary cultures of hippocampal neurons.

## RESULTS

### Localization of Shh in young hippocampus and cerebellum

**Fig. 1.**
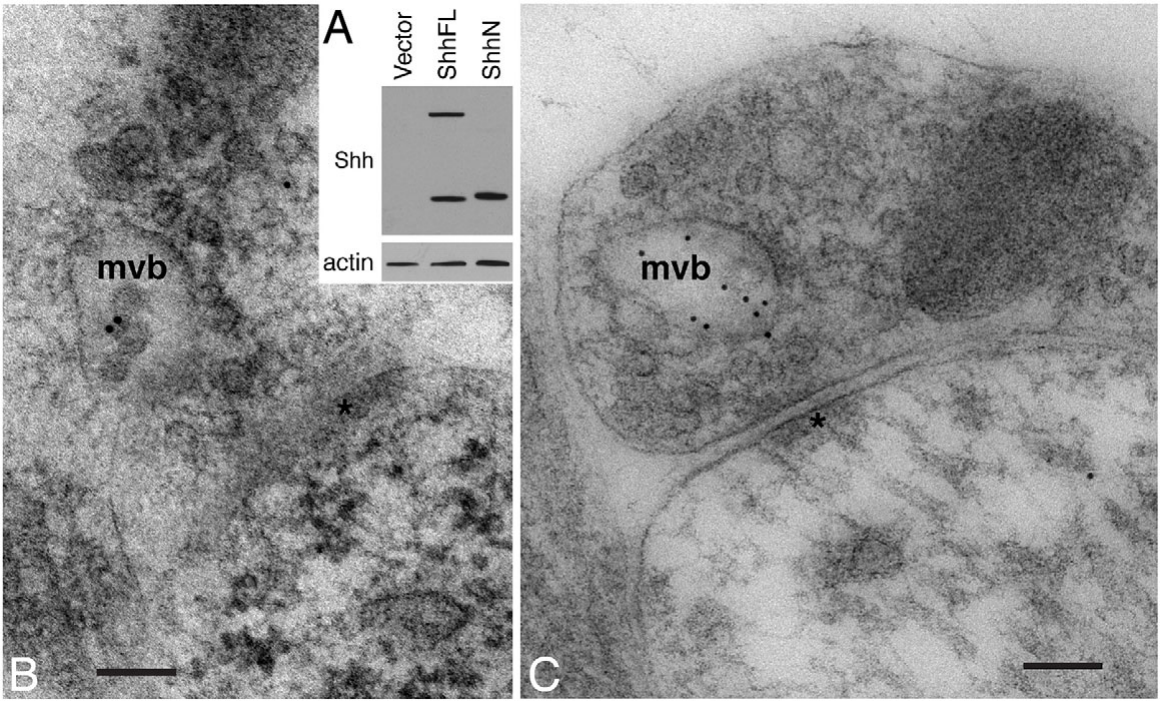
**Shh associates with the multivesicular bodies in growth cones of P2 rat hippocampus and cerebellum.** (A) Validation of the specificity of Shh 5E1 antibody. HEK293 cells were transfected with a full-length Shh construct (ShhFL), an N-terminal Shh fragment construct (ShhN), or a control vector. The cell lysates were analyzed by immunoblot using Shh 5E1 antibody. (B) In an axon terminal of the hippocampus, Shh immunogold particles are found in a multivesicular body (mvb). (C) In an axon terminal of the cerebellum, Shh immunogold particles are also found in an mvb. *, postsynaptic density. Scale bars: 100 nm.

We began by examining the location of Shh in the hippocampus and the cerebellum from postnatal day (P)2  rats. We performed immunoelectron microscopic analysis of these brain areas using Shh 5E1 antibody. The development and characterization of Shh 5E1 antibody has been described previously (Ericson et al., 1996). The antibody has been used for detecting Shh in various samples (Ericson et al., 1996; Cooper et al., 1998; Parra and Zou, 2010; Beug et al., 2011), including adult rat hippocampus (Petralia et al., 2011a). Immunoblot analysis of HEK293 cells expressing the full-length Shh or N-terminal fragment of Shh again confirmed the specificity of the antibody (Fig. 1A).

We observed Shh immunogold particles in multivesicular bodies (MVB) located in the axon terminals of synapses in the P2 hippocampus (Fig. 1B) and cerebellum (Fig. 1C). Because MVBs are believed to be a cellular source of exosomes (for review see Budnik et al., 2016), we focused on identifying exosomes positively labeled with Shh. However, surveying ∼400 electron micrographs in Shh-labeled P2 hippocampus and cerebellum, as well as numerous micrographs of Shh-labeled adult rat hippocampus (associated with a previous study; Petralia et al., 2011a), we did not find any Shh-labeled structures that were definitive EVs. The absence of unambiguously identifiable Shh-labeled EVs suggested two scenarios: (1) EVs are not morphologically preserved in our samples; and (2) in P2 hippocampus and cerebellum, Shh molecules, or the majority of them, are not carried by EVs.

**Fig. 2.**
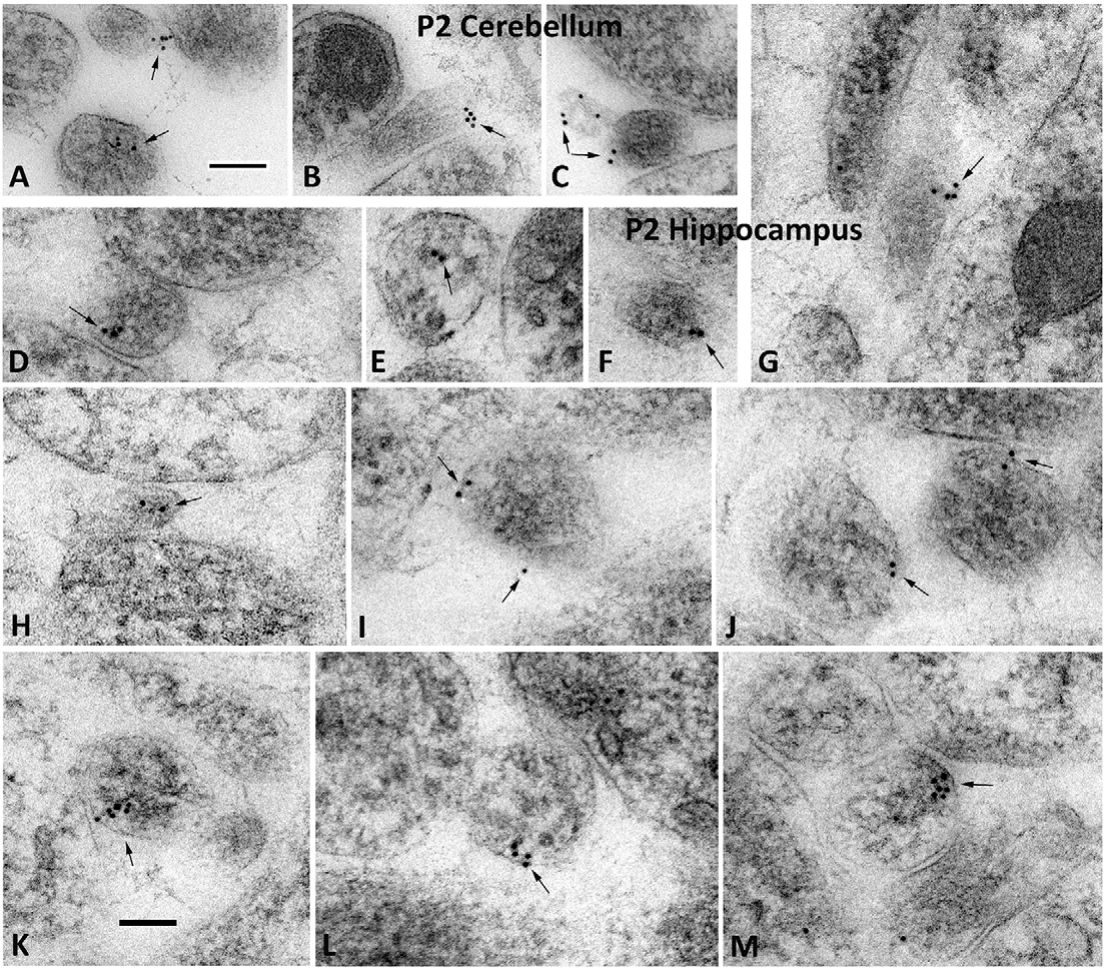
**Shh localizes to small processes in P2 cerebellum and hippocampus.** Immunogold particles for Shh (arrows) frequently associate with various small processes in the neuropil including thin neurites and filopodia in cross or oblique section view, showing that Shh localizes to small processes in P2 cerebellum (A-C) and hippocampus (D-M). Filopodia were identified based on their ultrastructural characteristics: thin, lacking microtubules, and usually absent organelles. In some cases, immunogold particles appear on the surface of a small vesicle in the cytoplasm (bottom in A; and D,E); whereas in other cases, the immunogold particles are located on the membrane surface (top in A; and B,C,F,G,I,J), which sometimes are located at a point of contact with an adjacent process (A,B,G,I; and J top structure). Labeled structures in J-M appear to be oblique sections through thin neurites (with possible profiles of oblique microtubule sections). Note how the gold labeling pattern in K-M appears the same, and may show labeling of a vesicle contacting the surface membrane; in M, the point of contact is adjacent to a cell junction – possibly an early synaptic contact. Scale bars: 100 nm. The control of omitting the primary antibody is shown in Fig. S1.

We switched our focus to elucidate the subcellular localization of Shh immunoreactivity and we found many examples of Shh-immunolabeled filopodia and thin neurites in the hippocampus and cerebellum of developing rats (Fig. 2). We identified thin neurites and filopodia based on their ultrastructural characteristics (Deitch and Banker, 1993; Petralia et al., 2015). Notably, clusters of immunogold labeling were found on the surface of these filopodia (Fig. 2A-C,F,G; Fig. S1) and thin neurites (Fig. 2I-M). In some cases, clusters of gold particles appeared to be associated with a pit or vesicle subjacent to the cell membrane (Fig. 2D,K-M). In most cases, the labeled surface of the process was in close contact with the surface of an adjacent filopodium or neurite (Fig. 2A-C,D,G,I-M). Therefore, in developing hippocampus and cerebellum, Shh molecules often localize in neurites and filopodia where they preferentially position at cell-to-cell contacts.

### Shh can be found inside as well as outside of cultured hippocampal neurons

**Fig. 3.**
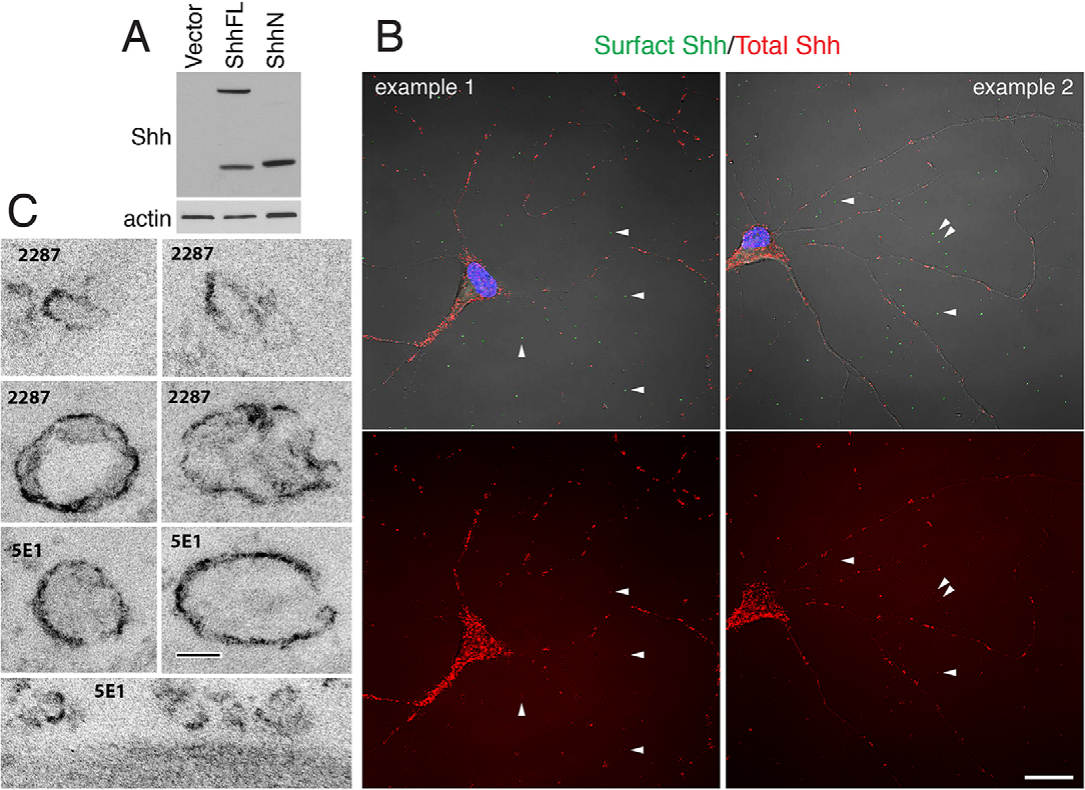
**Shh immunolabeled EVs are found in primary cultures of hippocampal neurons.** (A) Validation of the specificity of Shh 2287 antibody. ShhFL, a full-length Shh construct; ShhN, an N-terminal Shh fragment construct; Vector, control vector. (B) Representative confocal images of cultured hippocampal neurons labeled with two different Shh antibodies. Cell surface Shh was detected by labeling live neurons with one Shh antibody (2287, green); total Shh was detected by labeling fixed and permeabilized neurons with another Shh antibody (5E1, red). Note that in addition to punctate labeling on neurons, many Shh-labeled puncta are seen outside of and away from neurons (white arrowheads). Images are representative of data observed in at least four separate cultures. Scale bar: 20 µm. The controls for this labeling experiment are shown in Fig. S2. (C) Representative electron micrographs of Shh-labeled EVs found in primary cultures of hippocampal neurons. Neurons were fixed and labeled with either Shh 5E1 antibody or Shh 2287 antibody, followed by a biotinylated secondary antibody and avidin-biotin-peroxidase reagent before processing for electron microscopy. Dark surface labeling is the peroxidase-DAB reaction product. Notice that Shh-labeled EVs have a wide range of sizes. Scale bar: 100 nm.

Because EVs have been shown to be released by cultured cortical neurons (Fauré et al., 2006), we examined cultured hippocampal neurons and the distribution of Shh. In this set of experiments, we used two anti-Shh antibodies, Shh 5E1 antibody and Shh 2287 antibody (derived from mouse and rabbit, respectively). Specificity of Shh 2287 antibody is shown in Fig. 3A.

We labeled cultured neurons with one anti-Shh antibody before fixation and with another Shh antibody after fixation and membrane permeabilization. The former detected Shh molecules residing on the membrane surface, whereas the latter detected Shh throughout the cells. Overall, both Shh antibodies showed a similar labeling pattern (Fig. 3B, Fig. S2). In addition to Shh-immunolabeled puncta found on neurons, we repeatedly observed Shh puncta scattered outside of neurons (Fig. 3B). In order to identify the ultrastructure of these extracellular Shh-immunolabeled puncta, we carried out immunoelectron microscopic analysis. We found that, as exemplified in Fig. 3C, vesicular structures in a range of sizes (from 100-300 nm) exhibited notably dark Shh-immunoreactive products on their membrane surface. Therefore, in hippocampal neurons cultures, Shh can be found in EVs.

### Biochemical and functional analyses of Shh associated with extracellular vesicles

**Fig. 4.**
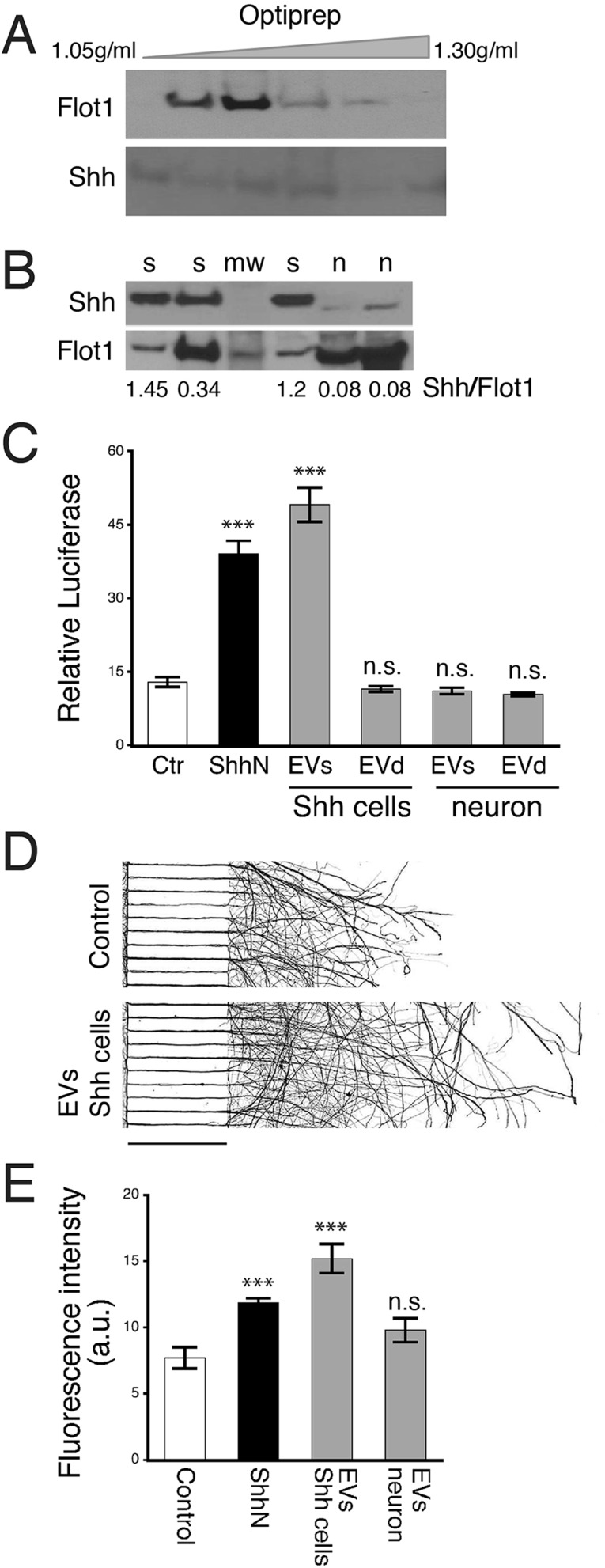
**Biochemical and functional measures of EVs isolated from cultured cells.** (A) EVs were isolated from the culture medium of a Shh-overexpressing cell line, ShhN-HEK293 (Shh cells). Immunoblots of density-fractionated samples show that Shh (detected by Shh 2287 antibody) is present, but is not concentrated in the Flot1-enriched exosome fraction. (B) EVs isolated from culture medium of hippocampal neurons were subjected to immunoblot analysis. Quantification of Shh by normalizing to Flot1 show significantly smaller amounts of Shh in neuron-derived EVs compared to EVs derived from Shh-overexpressing cells. s, Shh-overexpressing cells; n, neurons; mw, molecular weight markers. (C) Shh bioactivity was measured using Shh-Light2 assay. EVs from the Shh-overexpressing cell line (Shh cells) displayed high Shh activity, whereas EV-depleted samples (EVd) had no Shh activity. EVs from neurons were also assayed. No Shh bioactivity was detected in either EVs or EVd samples. Control (Ctr), culture medium from HEK293 cells; ShhN, culture medium from ShhN-HEK293 cells (5%). The assay was repeated three times. Data represented as mean±s.e.m.; ***P<0.001; n.s., not significant. (D) Representative images of neurons grown in a compartmentalized culture system for 6 days. EVs isolated from ShhN overexpressing cells were added to the soma compartment, and neurons were grown for one additional day. Neurons were immunolabeled with a neuronal marker, Tuj1. Images were inverted for better visualization of axons. Scale bar: 450 µm. (E) Quantitative analysis of Tuj1 fluorescence intensity in axons. The experiment was repeated four times. Data represented as mean±s.e.m.; ***P<0.001; n.s., not significant.

We next investigated Shh in biochemically purified EVs. We started by examining EVs purified from the culture medium of ShhN-HEK293 cells; a cell line overexpressing an N-terminal Shh fragment construct, ShhN (Chen et al., 2002a,b). Culture medium collected from this cell line is widely used as a source of bioactive ShhN (Chen et al., 2002a,b). Using immunoblotting, Shh protein was readily detected in EVs released from the Shh-overexpressing cell line (Fig. 4A). Density fractionation using Optiprep showed that Shh was not particularly enriched in the exosomes, but was instead, detected in EVs in a wide range of sizes and buoyant densities (Fig. 4A), a finding consistent with the immunoelectron microscopic images (Fig. 3C). To evaluate Shh activity in the purified EVs, we used a reporter cell-based assay, Shh-Light2 (Taipale et al., 2000). The purified EVs displayed a high level of Shh activity, whereas no Shh activity remained after depleting EVs (Fig. 4C, left). These results suggest that in the Shh-overexpressing cell line, the majority, if not all, of extracellular Shh is carried by EVs.

We also examined Shh in EVs isolated from cultured hippocampal neurons. Immunoblotting showed detectable Shh protein in these neuron-derived EVs; however, the amount of Shh in the neuron-derived EVs was significantly smaller than Shh present on the EVs derived from Shh-overexpressing cells (Fig. 4B; n, neurons; s, Shh-overexpressing cells). When we assessed Shh activity using the Shh-Light2 assay, Shh activity was undetectable in the hippocampal neuron-derived EVs (Fig. 4C, right).

Our prior experiments demonstrated Shh's ability to stimulate axon elongation (Yao et al., 2015); here, we assessed whether the neuron-derived EVs would facilitate axon growth. We added EV preparations to neurons growing in a compartmentalized culture system (Fig. 4D), and measured and compared axon lengths 1 day later. Strikingly, the EVs purified from the ShhN-overexpressing cells made neurons grow markedly longer axons, even longer than the parent medium did (Fig. 4D,E). EVs purified from hippocampal neurons, on the other hand, did not significantly stimulate axon growth (EVs neuron versus Control, *P*=0.088, *n*=4 cultures).

## DISCUSSION

The Shh signaling pathway has multiple functions in multiple tissues, from development to adulthood. While new functions of Shh signaling continue to be revealed and the mechanisms of Shh's transcellular signal transduction need to be further understood, it is well established that Shh molecules are lipophilic because of their dual-lipid modification. In various studies, EVs have been implicated in the intercellular transport of lipophilic Shh molecules (Tanaka et al., 2005; Liégeois et al., 2006; Matusek et al., 2014; Vyas et al., 2014). In this study, we focus primarily on investigating extracellular Shh in the hippocampus, where the Shh signaling pathway is known to be present (Traiffort et al., 1998; Beug et al., 2011; Petralia et al., 2011a,b; Yao et al., 2015).

We examined young hippocampal tissues, and also surveyed the young cerebellum – another brain area where Shh signaling is known to exist (Wechsler-Reya and Scott, 1999; Wallace, 1999; Petralia et al., 2012). In both the hippocampus and the cerebellum, we observed Shh molecules in the MVBs, which are generally considered to be a cellular source of small EVs exosomes (Budnik et al., 2016). However, we did not find any Shh-containing EVs despite intensive searching. Although we have noted circular structures of approximately the diameter of a typical exosome (<100 nm), none had the typical structure of an exosome as seen with electron microscopy (Fauré et al., 2006; Lachenal et al., 2011). In addition, without 3D reconstruction, it is difficult to distinguish whether these structures are other types of EVs (Tanaka et al., 2005; Muralidharan-Chari et al., 2009; Matusek et al., 2014) or a cross-section view of a filopodium or small neurite. Shh has been shown to be carried by nodal vesicular parcels in mouse embryos; it is not yet clear if a similar mechanism is used in the interstitial space of post-embryonic brains. Therefore, the question of whether the lack of identifiable Shh-containing EVs reflects the absence of EV-mediated Shh transport in intact brain tissues or if it is, instead, the result of technical limitations will have to await future investigation using technology such as multi-component 3D imaging.

Although our *in vivo* data do not address the question of extracellular Shh, we clearly observed that Shh molecules often localize in small thin neurites and filopodia. Within these structures, Shh molecules preferentially position near or at the membrane surface where it is either immediately adjacent to or in direct contact with the opposing membrane of a neighboring neurite (Fig. 2). The presence of Shh in filopodia and its preferred location at cell-to-cell contacts indicate that, perhaps similar to several other cell types (Rojas-Ríos et al., 2012; Bischoff et al., 2013; Sanders et al., 2013), Shh in the brain travels and spreads through filopodia.

In contrast to the *in vivo* observations in the intact brain tissues, we repeatedly discerned extracellular Shh-immunolabeled puncta scattered outside of neurons and their processes in primary cultures of hippocampal neurons (Fig. 3B). Further examination by immunoelectron microscopy revealed that these extracellular punctate structures are variously sized vesicles and vesiculate structures bearing Shh immunolabeling on their surface. Immunoblot analysis confirmed the presence of Shh in the EVs from cultured hippocampal neurons; however, the amount of EV-carried Shh from the neurons was significantly smaller than the amount of EV-Shh from the ShhN-overexpressing cells (Fig. 4C). Such a small amount of Shh was probably too low to activate the Shh pathway in the Shh-Light2 assay and neuronal axon elongation experiment.

A recent study reported that Hedgehog (Hh) associated with EV-released microparticles does not activate a canonical Hh signaling pathway in adipocytes (Fleury et al., 2016). It is noteworthy that activation of the Shh signaling pathway in the cells for both the Shh-Light2 and axon elongation assays requires Smoothened and the Gli transcription factor (Taipale et al., 2000; Yao et al., 2015). It has been shown that, in the cerebellum, the level of neuron-derived Shh determines the specific phenotype of neighboring glial cells; that high Shh-expressing Purkinje cells associate with Bergmann glia, whereas low Shh-expressing granule cells associate with astrocytes (Farmer et al., 2016). Therefore, it is plausible that in the hippocampus, filopodia-conveyed Shh and EV-carried Shh activate the pathway differently.

## MATERIALS AND METHODS

### Animals

Brain tissues from young rats (postnatal day 2; P2) were used for immunogold labeling. Timed pregnant female rats were used as the source of embryonic brains to establish cultures of hippocampal neurons. All animal procedures were approved by the NIA and the NIDCD Animal Care and Use Committee and complied with the NIH Guide for Care and Use of Laboratory Animals.

### Antibodies and DNA constructs

We used two anti-Shh antibodies in this study; a mouse monoclonal antibody, 5E1, obtained from Developmental Studies Hybridoma Bank, and a rabbit antibody obtained from Cell Signaling Technology (#2287). Both antibodies were raised against the N-terminus of Shh. To detect the EV marker flotilin1 (Flot1), a rabbit Flot1 antibody was used (Abcam, ab133497). Tuj1 antibody was from Convance.

The full-length and N-terminal fragment of Shh constructs were generously provided by Dr James K. Chen (Stanford University).

### Cell culture and transfection

Cultures of hippocampal neurons were prepared from embryonic day (E)18 rat brains as described (Kaech and Banker, 2006; Yao et al., 2015). The neurons were grown in Neurobasal medium supplemented with B27 (Invitrogen). For immunofluorescence and immunoelectron DAB microscopy, the neurons were grown on polylysine (1 mg ml^−1^)-coated glass coverslips. For immunoblotting, the neurons were grown in polylysine-coated plastic dishes. The age of the cultures used for experiments was between 8-13 days in culture. Human embryonic kidney (HEK) 293 cells obtained from ATCC were cultured according to the manufacturer's instructions and transfected using the FuGENE6 kit.

### Immunoelectron microscopy

For P2 hippocampal and cerebellar tissues, postembedding immunogold labeling was performed using previously published methods (Petralia and Wenthold, 1999; Petralia et al., 2010, 2011a,b). Briefly, following cryoprotection and embedding, tissue sections were further processed and embedded in Lowicryl HM-20 resin using a Leica AFS freeze-substitution instrument. After blocking, sections were incubated with primary antibody. Shh 5E1 antibody was used at 1:20-1:100. Following incubation with 10 nm gold-conjugated secondary antibody, the sections were stained with uranyl acetate and lead citrate. Sections were examined from two and three P2 rats for hippocampus and cerebellum, respectively. The control omitting the primary antibody is shown in Fig. S1.

For cultured hippocampal neurons, preembedding immunoperoxidase labeling was performed as described previously (Petralia and Wenthold, 1999; Petralia et al., 2010, 2011a, 2012; Lichnerova et al., 2015). After incubation with 3% normal goat serum (GS) and 3% BSA in Neurobasal medium, live neurons were incubated in 1% GS in Neurobasal medium containing either Shh 5E1 antibody (1:200) or Shh 2287 antibody (1:200) for 45 min at room temperature. Neurons were gently washed twice in Neurobasal medium and fixed in 4% paraformaldehyde for 20 min. Following washes in PBS, the neurons were labeled with biotinylated secondary antibody followed by the avidin-biotin-peroxidase reagent (Vectastain ABC kit) and the labeling was visualized using 3,3′-Diaminobenzidine (DAB) (Petralia and Wenthold, 1999; Petralia et al., 2010; Lichnerova et al., 2015).

For all electron microscopy methods, images were stored in their original formats and final images for figures were prepared in Adobe Photoshop; levels and brightness/contrast of images were minimally and evenly adjusted over the entire micrograph. Control sections (from two and three rats for P2 hippocampus and cerebellum, respectively) omitting the primary antibody showed only rare gold labeling.

### Immunocytochemistry and fluorescence microscopy

We labeled membrane surface Shh and total Shh in the same neurons by taking advantage of the species difference between Shh 2287 rabbit antibody and Shh 5E1 mouse antibody. Live neurons were incubated with Shh 2287 antibody (1:250) in Neurobasal medium containing 1% goat serum (GS) for 45 min at room temperature. The neurons were then fixed in 4% paraformaldehyde for 20 min, washed, and incubated with Alexa Fluor 488-tagged goat anti-rabbit secondary antibody. Following washes in PBS, the neurons were permeabilized in PBS containing 0.1% Triton X-100, blocked in 10% GS, and incubated with Shh 5E1 antibody (1:250) in PBS containing 1% GS. The neurons were then incubated with Alexa Fluor 568-tagged goat anti-mouse secondary antibody.

The labeled neurons were examined using a 40× or a 63× objective on a Zeiss LSM710 laser scanning confocal microscope. All images were acquired at a 1024×1024 pixel resolution and each image was an average of four scans at the same focal plane. The brightness, contrast and levels of the images were minimally adjusted (in Adobe Photoshop CS6) for those images presented. No additional digital image processing was performed.

### Immunoblotting

Cells were lysed in RIPA buffer (20 mM Tris-HCl, 150 mM NaCl, 1 mM EDTA, 1 mM EGTA, 1% NP-40, 2.5 mM sodium pyrophosphate, 1 mM sodium orthovanadate, and 1% sodium deoxycholate) containing protease inhibitors. The lysed cells were centrifuged at 10,000 ***g*** for 10 min at 4°C. The amount of total proteins in the cell lysates was estimated with a Pierce BCA protein assay kit (Pierce Biotechnology). Protein samples were separated by 4-20% Bis-Tris SDS-PAGE and transferred to nitrocellulose membranes. Following incubation with blocking buffer (5% nonfat milk and 0.05% Tween 20 in PBS), the membranes were incubated overnight at 4°C in the blocking buffer containing Shh 2287 antibody (1:500), Shh 5E1 antibody (1:150), Flot1 antibody (1:2000), or β-actin antibody (1:5000). The membranes were then washed (0.1% Tween 20 in PBS) and incubated with appropriate peroxidase-conjugate secondary antibodies. The proteins were visualized using a chemiluminescence kit from Pierce.

### Extracellular vesicle isolation

EVs were isolated as described previously (Théry et al., 2006; Lachenal et al., 2011). Briefly, 50 ml of primary hippocampal neuron culture medium was centrifuged at 500×***g*** for 10 min to remove dead cells, then the supernatant was centrifuged at 2300×***g*** for 10 min to remove cell debris and the supernatant was stored at −20°C. Once thawed (at room temperature), the medium was centrifuged at 120,000×***g*** for 2.5 h (SW28 rotor K=246). Following this the supernatant was removed for use as an EV-depleted control and the pellet was suspended in 3.5 ml sterile filtered PBS and centrifuged again at 120,000×***g*** for 2.5 h (SW55 rotor K=48). The supernatant was carefully removed and the pellet containing EVs was re-suspended in either lysis buffer for protein quantification or PBS for functional assays. The density of the vesicles was determined by loading them on top of an Iodixanol gradient (40%, 30%, 15%, 10%, 5%) and centrifuging at 150,000×***g*** for 16 h (SW41 rotor), and the different fractions were collected, diluted with water in a ratio of 1:6 and centrifuged at 120,000×***g*** for 2 h. Then the supernatant was carefully removed and the pellet was re-suspended in lysis buffer for further analysis.

### ShhN-containing conditioned medium

HEK293 cells that overexpress ShhN were kindly provided by Dr James K. Chen (Stanford University). We prepared ShhN-conditioned medium from ShhN-overexpressing HEK293 cells exactly as described (Chen et al., 2002a,b). Control HEK293 cell-conditioned medium was prepared from HEK293 cells that do not express the ShhN. Throughout this study, we use ‘ShhN-medium’ to refer to Shh-N-conditioned medium, and ‘control-medium’ to refer to control HEK293 cell-conditioned medium.

### Cell-based Shh activity assay

Shh activity was measured using Shh-Light2 cells, a clonal NIH3T3 cell line stably expressing Gli-dependent firefly luciferase and constitutive *Renilla* luciferase reporters (Taipale et al., 2000). Shh-Light2 cells were cultured in DMEM containing 10% bovine calf serum, 0.4 mg/ml G-418, and 0.15 mg/ml Zeocin. When confluent, the cells were treated with EV preparations being tested, ShhN-medium (5%, as a positive control), or control-medium (5%, as a negative control) in DMEM containing 0.5% bovine calf serum. After an additional 30 h in culture, the cells were lysed and assayed using the Dual Luciferase Assay kit (Promega) according to the manufacturer's protocol.

### Compartmentalized axon growth assay

Dissociated hippocampal neurons were grown in microfluidic chamber devices (Xona Microfluidics) as previously described (Taylor et al., 2005; Yao et al., 2015). After 6 days in culture, ShhN or EV samples were added to the somatodendritic compartment, and 24 h later, the neurons were fixed and labeled with neuronal marker Tuj1.

### Statistics

ImageJ (NIH) and KaleidaGraph (Synergy) software were used for measurements and statistics. Statistical comparisons were performed by using Student's *t*-test. All results are expressed as mean±s.e.m. Significance is defined as *P*<0.05.
